# Kinematic descriptors of arm reaching movement are sensitive to hemisphere-specific immediate neuromodulatory effects of transcranial direct current stimulation post stroke

**DOI:** 10.1038/s41598-024-62889-0

**Published:** 2024-05-25

**Authors:** Justine Lowenthal-Raz, Dario G. Liebermann, Jason Friedman, Nachum Soroker

**Affiliations:** 1 Physical Therapy Department, Loewenstein Rehabilitation Medical Center, Ra’anana, Israel; 2 Neurological Rehabilitation Department, Loewenstein Rehabilitation Medical Center, Ra’anana, Israel; 3 Physical Therapy Department, Stanley Steyer School of Health Professions, Faculty of Medicine, Tel-Aviv University, Tel Aviv, Israel; 4 Faculty of Medicine, Tel-Aviv University, Tel Aviv, Israel; 5 Sagol School of Neuroscience, Tel Aviv University, Tel Aviv, Israel

**Keywords:** Neuroscience, Biomarkers, Neurology

## Abstract

Transcranial direct current stimulation (tDCS) exerts beneficial effects on motor recovery after stroke, presumably by enhancement of adaptive neural plasticity. However, patients with extensive damage may experience null or deleterious effects with the predominant application mode of anodal (excitatory) stimulation of the damaged hemisphere. In such cases, excitatory stimulation of the non-damaged hemisphere might be considered. Here we asked whether tDCS exerts a measurable effect on movement quality of the hemiparetic upper limb, following just a single treatment session. Such effect may inform on the hemisphere that should be excited. Using a single-blinded crossover experimental design, stroke patients and healthy control subjects were assessed before and after anodal, cathodal and sham tDCS, each provided during a single session of reaching training (repeated point-to-point hand movement on an electronic tablet). Group comparisons of endpoint kinematics at baseline—number of peaks in the speed profile (NoP; smoothness), hand-path deviations from the straight line (SLD; accuracy) and movement time (MT; speed)—disclosed greater NoP, larger SLD and longer MT in the stroke group. NoP and MT revealed an advantage for anodal compared to sham stimulation of the lesioned hemisphere. NoP and MT improvements under anodal stimulation of the non-lesioned hemisphere correlated positively with the severity of hemiparesis. Damage to specific cortical regions and white-matter tracts was associated with lower kinematic gains from tDCS. The study shows that simple descriptors of movement kinematics of the hemiparetic upper limb are sensitive enough to demonstrate gain from neuromodulation by tDCS, following just a single session of reaching training. Moreover, the results show that tDCS-related gain is affected by the severity of baseline motor impairment, and by lesion topography.

## Introduction

Upper limb (UL) paresis is a major disabling consequence of stroke. Standard rehabilitation programs, based mostly on provision of physical and occupational therapy, often lack the intensity needed for promotion of significant motor recovery by use-dependent plasticity, and the impact of treatment is moderate at best^1–5^. Modulation of motor-cortex excitability by transcranial direct current stimulation (tDCS), in conjunction with rehabilitation treatment, was shown to have an added value relative to similar treatment without tDCS. TDCS elicits reversible shifts in cortical excitability depending on polarity, intensity and duration of stimulation^6^. Anodal and cathodal tDCS (A-tDCS, C-tDCS) applied over cortical motor regions, have excitatory and inhibitory effects, respectively^7,8^. Systematic reviews of physiological and functional effects in therapies employing tDCS report on different effect sizes of such interventions^6,9–12^.

A predominant practice in clinical tDCS research is to apply excitatory A-tDCS over motor regions of the damaged hemisphere. This approach is based on the assumption that such stimulation facilitates adaptive plasticity and functional re-mapping in peri-lesional cortical regions which play a crucial role in the recovery process^13^. Excitatory A-tDCS of the damaged hemisphere is often applied in conjunction with inhibitory C-tDCS of the contra-lesional motor cortex^8^, under the assumption that by the effect of reciprocal inter-hemispheric inhibition, the contra-lesional hemisphere exerts a negative effect on recovery processes based on peri-lesional cortex reorganization^14^. However, the idea that inter-hemispheric inhibition plays a negative role in motor recovery was questioned in recent years^15,16^, and studies have demonstrated that some patients may benefit from excitatory A-tDCS applied over the contra-lesional rather than the ipsi-lesional hemisphere^17^. A refined theoretical account of neuro-modulation (the bimodal balance-recovery model^18^), proposes that in patients with extensive unilateral damage to the motor system, ineffective cortico-spinal output from the damaged hemisphere may leave the intact non-crossing motor pathways descending from the non-affected hemisphere as the sole mediators of cerebral control over spinal motor neurons. In such cases, excitatory A-tDCS may positively affect motor recovery when applied over the motor system of the contra-lesional hemisphere^17,19,20^. Yet, given the large response variability in non-invasive brain stimulation (NIBS) methods, including tDCS^21–23^, it remains unclear how to decide what hemisphere should be selected for excitatory stimulation in each individual stroke patient.

Past studies usually examined the effectiveness of treatments incorporating tDCS using clinical measures of motor performance that cannot reliably distinguish between functional improvement gained by ‘restitution’ (‘true recovery’, reflected in basic constituents of the motor act) and functional improvement reflecting ‘compensation’ (i.e., the learned adoption of compensatory strategies aimed primarily to lessen dependency on help from others in activities of daily living). Analysis of movement kinematics offer a more proper way for evaluation of treatments aimed to facilitate restitution, being less sensitive to the effects of compensatory strategies^24–26^. Measures of end-point kinematics during point-to-point reaching movements, like smoothness (number of peaks in the speed profile; NoP), precision (e.g., deviation of the hand path from the straight line in point-to-point movement; SLD) and movement time (MT), quantify how well the motor task is performed at the hand level. Kinematic analysis of planar reaching movements in the current work is derived from a maximal smoothness assumption, i.e., the minimum-jerk model^27^. According to this model, point-to-point hand movements on a horizontal plane are normally characterized by nearly straight-line paths and bell-shaped speed profiles with a single peak. The peak represents the transition between acceleration and deceleration phases during the transport of the hand to the target. In general, the more peaks there are in the speed profiles, the less smooth and more segmented the movement is. Under the minimum-jerk model, a straight-line path is not a goal of the motor plan but rather an optimal solution implemented by the brain to perform maximally smooth movements. Studies of development and recovery from neural injury suggest that smoothness results from learned coordination of muscle activations^28,29^. Analysis of movement kinematics in carefully designed tasks, with reference to the basic aspects of smoothness, precision and speed, can thus be used as a sensitive measure of treatment-related dynamics representing true motor recovery (i.e., gains that reflect restitution of basic motor control, rather than better overall functionality achieved by the use of compensatory strategies)^30,31^. “Improvement” in the current context would be getting closer to optimal values in terms of the above kinematic descriptors, as shown by healthy controls.

Beyond the impact of abovementioned methodological issues, response variability of NIBS is thought to relate to variance in lesion patterns and corticospinal reserve. Lesion extent and topography may dictate targeting cortical regions within the premotor rather than primary motor cortex^20^ or stimulation of the contralesional rather than the ipsilesional hemisphere, in accord with the bimodal balance-recovery model^18^. This state of affairs makes it important to search for biomarkers that can point to the optimal stimulation target in different stroke patients. Evidence from the motor-learning literature points to the predictive value of performance changes obtained at the very initial stages of motor learning, even after a single training session^32^. Encouraged by the possibility of single-session effects to inform on future performance, we asked whether a single session of tDCS, applied in conjunction with arm reaching training, is likely to induce a measurable change in movement kinematics. If such effect is obtained and found to differ following stimulation of the motor cortex in the lesioned and non-lesioned hemispheres, it may serve the desired role of a biomarker indicating in a given patient which hemisphere (ipsi-lesional, contralesional) is preferred as a target for transcranial direct-current excitatory (anodal) stimulation. We hypothesized that excitatory A-tDCS applied on ipsi-lesional M1 will induce an improvement in stroke patients with milder paresis (relatively more preserved integrity of the motor network), whereas A-tDCS applied on the contralesional M1 may benefit patients with more severe paresis (indicating greater damage to the integrity of the motor system with markedly reduced corticospinal output from the lesioned hemisphere, and more supraspinal control being conveyed in homolateral manner). For a wide exposition of empirical data and theoretical derivations of the bimodal balance-recovery model of neuroplasticity see ^16,17,19,20,33,34^.

## Methods

### Participants

Forty-five subacute stroke patients hospitalized in the Department of Neurological Rehabilitation at the Loewenstein Rehabilitation Medical Center (LRMC; Raanana, Israel) were recruited for the study on the basis of the following inclusion criteria: first-ever ischemic or hemorrhagic stroke confirmed by CT or MRI scan, time after stroke onset 3–10 weeks, right handedness, age range 25–80, stable clinical and metabolic state, residual movement in the hemiparetic upper limb enabling performance of the experimental task (point-to-point short-trajectory movement in the horizontal plane). Exclusion criteria: psychiatric or neurological disorders in the past, craniotomy, seizures, cardiac pacemaker. Note that we did not stratify patients on the basis of severity, as the main aim of this study was to test the likelihood of obtaining differential hemispheric effects for just a single session of excitatory tDCS in patients with less versus more severe motor impairment. Thus, we recruited patients who answered the above inclusion criteria, who had different levels of upper limb impairment and we tested the correspondence between tDCS effects and (1) the level of clinical impairment (as reflected in the baseline FMA-UE score) and (2) the extent of damage to regions of the motor network (as reflected by the percent of damage to regions of interest in the normalized brain scans), as explained later. Forty-one healthy subjects were recruited for a control group. The study was approved by the LRMC Institutional Review Board/Ethics committee, in accordance with the WMA Declaration of Helsinki (approval number LOE-10-0029) and all the participants signed a written informed consent. All the subjects underwent a preliminary evaluation where some exhibited difficulty performing the many repetitions needed and some expressed uneasiness or concern about the electrical stimulation and had to be excluded. In the end, 35 healthy subjects (25 females, 10 males; age range 25–75; self-reported health status) and 35 stroke patients (13 females, 22 males; age range: 26–77; FMA range: 11–62) were included in the study (see Table 1 for group details).

**Table 1:** Descriptive data of the sample population.

	Healthy subjects (n = 35)	Stroke patients (n = 35)
Age (years)	55 ± 5 (25–75)	60 ± 11 (26–77)
Sex	25 Females,10 Males	13 Females, 22 Males
Fugl–Meyer score	–	46.6 ± 16.3 (11–62)
Hemiparesis side	–	Right-18, Left-17
Time after stroke onset (days)	–	47 ± 23

Mean ± SD (range) values.

### Study design

A crossover single-blinded experimental design was used, where each subject underwent three sessions with a washout period of a minimum of 48 h. In each session subjects were tested in one of three stimulation conditions in a random order: excitatory anodal tDCS on the ipsi-lesional M1 (A-tDCS); inhibitory cathodal tDCS on the ipsi-lesional M1 (C-tDCS); sham condition (S-tDCS, using the A-tDCS montage). In each session, the anode and cathode were placed on opposite hemisphere M1 hand area—C3 and C4 electrode sites in the 10–20 system—such that C-tDCS is actually a condition in which the contralesional M1 receives excitatory anodal stimulation.

### Motor task

**Figure 1:**
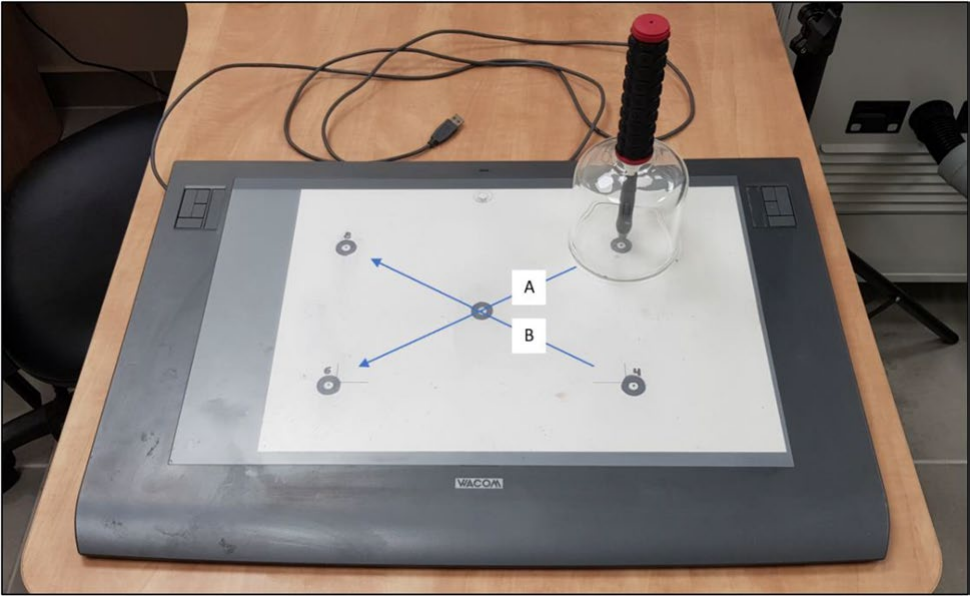
The handle with the stylus on the graphics tablet used in the experiment, with start and end points (in the case of right-hand performance, movements in directions A and B involve elbow flexion and extension, respectively). Starting points for right-hand movements—far right, near right. Starting points for left-hand movements—far left, near left.

Subjects performed point-to-point diagonal movements in the horizontal plane on a graphics tablet (Intuos-3, Wacom Ltd, USA) within a working area of 30.5 × 45.7 cm). To facilitate grasping by patients with spastic hands, the stylus was connected to a specially designed wide handle of a bell-shaped transparent glass, enabling smooth slide on the surface of the tablet without obscuring the stylus tip and the target points (Fig. 1). The two-dimensional location of the tip of the stylus was recorded with an accuracy of 100 lines/mm at a sampling rate of 200 Hz. The tablet was interfaced to a PC where data were collected using a custom-made code written in MATLAB (v8.6, MathWorks Ltd., Natick, Ma, USA). Off-line processing was also performed using MATLAB routines. The raw tablet data were filtered using smoothing splines with knots every 6 samples. Subjects sat at a table at waist height, stroke patients had their trunk strapped to the chair to prevent forward leaning as a compensatory movement.

Movements were performed in two diagonal directions (Fig. 1), one involving primarily elbow flexion and the other primarily elbow extension (for the right UL—from far-right point to near-left point, and from near-right point to far-left point, respectively; for the left UL—from far-left point to near-right point, and from near-left point to far-right point, respectively). After reaching the target point, the subject had to pick up the handle, bring it back to the starting point and start a new trial upon hearing an auditory cue. Movements in each direction were repeated in blocks of 15 repetitions. Subjects were encouraged to continue with the task until its completion, irrespective of the quality of their performance, and no feedback was provided throughout the experiment time. Control subjects performed the task with the dominant right hand and stroke patients (all right handers) with the hemiparetic upper limb (18 right, 17 left).

The instruction was to perform the task “as fast and as accurately as possible”. Each session lasted 60–90 min (patients were slower than healthy subjects), and comprised four stages, with a 5 min break between stages and a 30 s rest between blocks. The four stages were as follows:

- *Familiarization* One block of 15 repetitions per movement direction.
- *Baseline* Four blocks of 15 repetitions per movement direction (120 trials in total).
- *Training (under tDCS)* Six blocks of 15 repetitions per movement direction (180 trials).
- *Post-test* Four blocks of 15 repetitions per movement direction (120 trials).

### Brain stimulation

Phoresor II Auto Model stimulator PM 850 (IOMED®, USA) was used for tDCS delivery via two 35 cm^2^ electrodes (7 × 5 cm). In real stimulation mode, a 1 mA current was administered during the first 20 min of the training stage in each session. In the sham mode the current was delivered only at the onset (30 s ramp up, 60 s stimulation at 1 mA, 30 s ramp down) then terminated. In all modes, the intensity was ramped up and ramped down for 30 s each.

### Primary outcomes

The gains (change from baseline to post-training performance) in three features of arm movement kinematics served as primary outcomes: (1) *Movement time* (MT, s)—the time interval between initiation and termination of the point-to-point movement, defined as the first and last times the speed (i.e., the tangential velocity profile) was more than 5% of the peak speed, for start and stop, respectively; (2) *Number of peaks* (NoP, counts)—in the speed profile, used as a measure of movement smoothness. If the distance between peaks was less than 40 ms, the second peak was not included in the count, as we assumed it to be spurious (given that latencies of monosynaptic reflexes in the human upper limb are in the range of 20–40 ms); (3) *Straight-line deviation* (SLD, cm)—a spatial error variable computed as the mean absolute error from the straight line connecting the initial and final points in the horizontal plane. In this measure of path accuracy, zero difference from the shortest line between two points means no error.

### Statistical analysis

Statistical analyses were performed using SPSS version 24.0 (IBM Corp, Armonk, NY). Kinematic measures were analyzed separately for flexion and extension movement directions, given the typical asymmetry of stroke-related muscle hypertonia revealed by elbow flexors and extensors. Given the limited statistical power inherent to our cohort size, the almost equal numbers of stroke patients performing the task with each hand, and the fact that our principal research question is answered mainly by within-group rather than between-groups analyses, we considered the average stroke-group performance as a reflection of movement kinematics of the hemiparetic upper limb, irrespective of whether hemiparesis affected the dominant or the non-dominant hand. The impact of stimulation mode on each kinematic measure was assessed by comparing post-test and pre-test (baseline) results. As an a priori decision, median values were computed for performance in each block of trials, to reduce possible effects of outliers. Later, these values were used to calculate a participant’s mean performance level in each experimental stage and stimulation condition, and these average values were used to compute stimulus-associated gains by comparing the mean value at the baseline stage and the mean value at the post-training stage. Given the dependence of NoP on movement duration and speed (i.e., longer and slower movements inherently increase the chance of having more peaks), we made no assumptions about its distribution. Thus, nonparametric testing was implemented to assess differences in NoP. The other features—MT and SLD—were assumed to be normally distributed, and we confirmed that using the Shapiro–Wilk test and applied parametric tests in their analysis. The statistical analyses did not include trials in which subjects did not reach the end target. To avoid confusion, note please that stimulation *gain* is expressed by a positive value, representing the extent of *improvement* obtained in the training session, though in essence improvement here means smaller NoP and SLD and shorter MT.

An outlier analysis was performed using Mahalanobis distances, with alpha level set at 5%. Subjects were eliminated from the group analysis when they presented highly unusual outcome scores (deviating by more than 2SD from the group mean) on the three kinematic variables, in all stimulation conditions. For flexion movements, 35 stroke patients and 32 healthy controls remained while for extension movements, 35 stroke patients and 34 healthy controls were eventually included in the group analysis.

For comparison between healthy subjects and stroke patients at baseline, the non-parametric Mann–Whitney U test was used for the NoP variable, where improvement in movement quality (smoothness) is expressed by reduction of NoP. Student’s t-test was used for MT and SLD variables. The analyses were based on the baseline (pre stimulation) stage of the first stimulation session in each subject. A mixed-design ANOVA with Group (stroke, control) as a between-subjects variable and Stimulation mode (A-tDCS, C-tDCS, S-tDCS—all referring to the lesioned hemisphere in the stroke group and to the left hemisphere, contralateral to the examined dominant right hand, in the group of healthy controls) as a within-subjects variable, was used to assess differences in stimulation effects between patients and healthy subjects. For within-group analysis, Friedman’s ANOVA and Wilcoxon signed-rank test were used for the NoP variable and ANOVAs were used for the MT and SLD variables. Bonferroni corrections were used in multiple pairwise comparisons. Spearman’s correlations were used to assess the relationship between stimulus effects and initial motor impairment as reflected in patients’ scores in the Fugl-Meyer assessment scale for the upper extremity^35^.

### Lesion analysis

**Figure 2:**
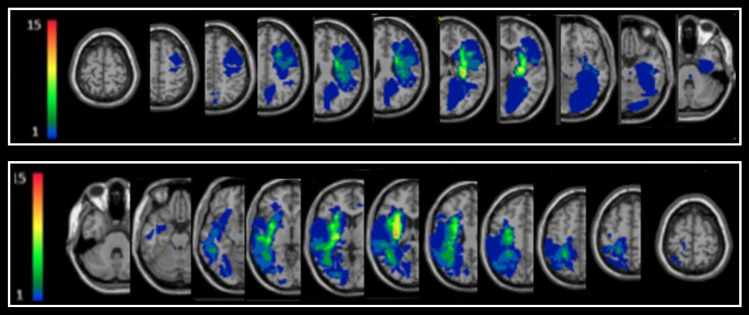
Lesion overlay maps of left- (n = 17) and right-hemisphere damaged (n = 16) patients—top and bottom rows, respectively. Representative normalized slices (out of 90 normalized slices) are displayed in radiological convention (right hemisphere on left side and vice versa), with warmer colors indicating greater lesion overlap (units: number of patients with lesion in the colored region).

Delineation of lesion boundaries was done manually on digitized follow-up brain CT or MRI scans, dated on average 40 days post stroke onset, by a physician experienced in analyzing neuro-imaging data, who was blinded to the stroke patients’ information (co-author NS). Lesion analyses were performed with the Analysis of Brain Lesions (ABLe) module implemented in MEDx software (Medical-Numerics, Sterling, VA, USA). ABLe characterizes brain lesions in MRI and CT scans of the adult human brain by spatially normalizing the lesioned brain into the Montreal Neurological Institute (MNI) standard template. It reports tissue damage in the normalized brain using an interface to the *Talairach Daemon* (San Antonio, Texas), *Automated Anatomical Labeling* (AAL), *Volume Occupancy Talairach Labels* (VOTL), and the *Stereotactic White Matter* (sWM) atlases^36–39^. In the current study, we quantified the amount of tissue damage within each structure/region of the AAL and sWM atlases as described earlier^40^. Registration accuracy of the scans to the MNI template was 93.9 ± 1.6 among right hemisphere damaged (RHD) patients and 93.6 ± 1.9% among left hemisphere damaged (LHD) patients. Normalized lesion overlay figures for the patient groups show a typical distribution of damage mainly in MCA territory (Fig. 2).

### Voxel-based lesion-symptom mapping (VLSM)

VLSM^41^ was used to identify voxels of the normalized brain where the existence of damage had a significant impact on the ‘gain’ obtained in each of the three stimulation conditions (A-tDCS, C-tDCS and S-tDCS) as reflected in measures of arm kinematics (NoP, MT, SLD). The term ‘gain’ means here smaller NoP, shorter MT and smaller SLD in the post- compared to the pre-stimulation test. This comparison is done—separately for each voxel—between the average gain obtained by subjects with damage to that voxel and the average gain obtained by subjects without damage to that voxel. The subjects with and without damage to voxel_[x,y,z]_ are likely to be different from the subjects with and without damage to voxel_[x′,y′,z′]_ if the two voxels are distant one from another and receive their blood supply from different branches of the arterial tree. Note that the ‘gain’ is analyzed here separately for each stimulation condition, irrespective of whether this ‘gain’ reached or did not reach significance in the analysis of variance testing for *differences* between the three stimulation conditions (Table 2). The statistical significance of differences in stimulation ‘gain’ between stroke patients with and without damage in a given voxel, was calculated using the Mann–Whitney test (due to ordinal [NoP] or non-normally distributed variables [MT, SLD]). Due to the relatively small cohorts of LHD and RHD patients, the left hemisphere was flipped to the right such that VLSM ignored lesion side. Only voxels damaged in at least 20% of the patients (n = 7) were tested, and at least 10 adjacent voxels had to show a statistically significant impact of damage on stimulation gain for a cluster of voxels to be reported. To correct for multiple comparisons, voxels with values exceeding a false discovery rate (FDR) of p < 0.05 were considered significant. When VLSM results did not survive the FDR correction, we used a more lenient criterion of p < 0.005, reflecting z ≥ 2.6 (for a similar approach see^42,43^). This information is provided under the assumption that VLSM points to brain regions likely implicated in movement kinematics in such cases, though the association has to be verified by further investigation. We report anatomical regions containing voxel clusters in which the existence of damage affected patients’ baseline scores (Table 3) and anatomical regions containing voxel clusters in which the existence of damage affected the stimulation gain relative to patients who were not affected in these voxels (Table 4). The maximum z-score is reported for each cluster of contiguous above-threshold voxels. Since there may be multiple voxels with this maximum z-score in the cluster, we report the coordinates of the voxel that is most superior, posterior, and left in its location within the cluster (the centroid of the cluster is not reported as it may not have the highest z-score value and it may not be an above-threshold voxel). The AAL atlas for gray matter and the sWM Atlas ^36–39^ were used to identify the brain structures in which the significant clusters were located.

**Table 2:** Stimulation gain in A-tDCS, C-tDCS and S-tDCS as reflected in movement kinematics.

Variable	Movement direction	Stimulation mode	Control n = 32, 34* Mean (SD)	Stroke n = 35 Mean (SD)	Stimulation mode	Group	Stim mode × group	Post-Hoc
NoP	Flexion	A-tDCS	0.29 (0.78)	0.82(1.28)	F(2,130) = 4.61 *p = 0.012	F(1,65) = 0.95 p = 0.7	F(2,130) = 2.92 *p = 0.05	A-tDCS vs S-tDCS *p = 0.017
		C-tDCS	0.28 (0.9)	0.36 (1.46)				
		S-DCS	0.17 (0.62)	− 0.23 (1.9)				
	Extension	A-tDCS	0.16 (0.56)	0.92 (2.64)	F(2,128) = 2.64 p = 0.07	F(1,65) = 0.73 p = 0.3	F(2,128) = 1.33 p = 0.2	–
		C-tDCS	0 (0.61)	0.38 (2.07)				
		S-tDCS	− 0.02 (0.43)	− 0.15 (1.61)				
MT	Flexion	A-tDCS	0.06 (0.18)	0.27 (0.48)	F(2,130) = 3.38 *p = 0.04	F(1,65) = 0.72 p = 0.3	F(2,130) = 2.54 *p = 0.05	–
		C-tDCS	0.07 (0.23)	0.11 (0.5)				
		S-tDCS	0.04 (0.14)	− 0.09 (0.61)				
	Extension	A-tDCS	0.08 (0.19)	0.26 (0.64)	F(2,128) = 2.51 p = 0.07	F(1,64) = 2.52 p = 0.1	F(2,128) = 0.67 p = 0.5	–
		C-tDCS	0.06 (0.24)	0.15 (0.69)				
		S-tDCS	0 (0.2)	0 (0.55)				
SLD (cm)	Flexion	A-tDCS	− 0.01 (0.1)	0.07 (0.16)	F(2,130) = 2.05 p = 0.1	F(1,64) = 1.88 p = 0.1	F(2,‏130‏) = 2.‏37 p = 0.‏1‏	–
		C-tDCS	− 0.01 (0.07)	− 0.03 (0.27)				
		S-tDCS	0 (0.1)	0 (0.22)				
	Extension	A-tDCS	0.002 (0.13)	0.04 (0.24)	F(2,‏128‏) = 1.52 p = 0.‏12	F(1,64) = 2.52 p = 0.1	F(2,‏128‏) = 1.14 p = 0.‏3	–
		C-tDCS	− 0.001 (0.08)	0.01 (0.26)				
		S-tDCS	0.02 (0.07)	0.5 (2.42)				

*NoP* number of peaks, *MT* movement time, *SLD* straight-line deviation, *A-tDCS* Anode placed over the hand area of the primary motor cortex in the lesioned hemisphere, *C-tDCS* Cathode placed over the hand area of the primary motor cortex in the lesioned hemisphere (homologous region in the non-lesioned hemisphere is under the anode), *S-tDCS* Sham stimulation.

*Number of healthy subjects analyzed was 32 and 34, in flexion and extension movements, respectively. The different numbers stem from exclusion of subjects with highly unusual performance, deviating by more than 2 SD from the group average on the 3 kinematic variables, in all stimulation conditions, as evidenced using Mahalanobis distances, with alpha level set at 5%. Stimulation gain was calculated by comparing in each patient post- and pre-stimulation scores, then averaging the individual gains to obtain a group mean and standard deviation. Note that the gain is expressed by a positive value, representing the extent of improvement obtained in the training session, though in essence improvement here means smaller NoP and SLD and shorter MT.

**Table 3:** Significant lesion effects on movement kinematics at baseline**.**

Kinematic variable	Structure	Z-value	x	y	z	Voxels	%Area
SLD—E	SCR	7.14	− 28	− 16	20	596	64.50
	Insula	7.54	− 32	− 20	20	576	31
	SLF	5.05	− 32	− 10	22	463	8.32
	Precentral G	3.78	− 40	0	26	451	12.79
	Putamen	5.43	− 28	− 10	10	330	32.71
	Rolandic operculum	4.49	− 32	− 26	18	315	31.82
	EC	6.49	− 30	− 18	16	294	5.28
	Poscentral G	3.23	− 44	− 12	30	264	6.78
	ACR	4.75	− 24	22	18	216	24.97
	PLIC	6.28	− 26	− 20	16	212	44.44
	Inferior frontal G_PT_	3.23	− 42	14	22	183	17.63
	Supramarginal G	3.17	− 48	− 42	26	86	6.85
	ALIC	3.30	− 22	− 4	18	77	19.64
	Heschl G	4.00	− 32	− 24	14	76	33.78
	Caudate	4.77	− 20	− 18	20	76	7.90
	Thalamus	4.42	− 20	− 16	8	67	6.09
	RLIC	5.35	− 26	− 24	16	66	21.22
	Superior temporal G	3.17	− 40	− 4	− 8	60	2.61
	PCR	6.67	− 28	− 22	22	59	13.23
	IFO	4.49	− 34	− 2	− 4	44	0.79
	Middle temporal G	2.89	− 48	− 12	− 22	37	0.75
	Middle frontal G	3.23	− 30	16	32	33	0.68
	Angular G	2.10	− 44	− 46	22	31	2.64
	SFO	4.08	− 20	− 8	20	31	0.56
	Inferior frontal G_PT_	3.23	− 36	18	24	30	1.19
	Middle temporal G	3.17	− 50	− 46	22	20	0.40
	Fusiform G	2.89	− 24	− 78	− 12	14	0.61
	Middle occipital G	2.89	− 28	− 74	− 2	14	0.43
SLD—F	Precentral G	3.78	− 36	− 6	38	517	15.84
	SCR	4.81	− 26	− 10	26	358	38.74
	Rolandic operculum	3.23	− 42	2	12	302	9.25
	Postcentral G	3.78	− 36	− 12	38	283	8.67
	SLF	4.08	− 34	− 18	28	279	34.23
	Inferior frontal G_PO_	3.78	− 44	14	18	192	5.88
	Insula	3.77	− 32	− 4	12	190	5.82
	PLIC	5.20	− 22	− 20	10	154	32.29
	Thalamus	4.57	− 20	− 22	6	95	2.91
	EC	3.77	− 30	0	10	54	12
	Caudate	3.55	− 18	− 16	20	49	1.50
	RLIC	3.88	− 26	− 18	0	45	14.47
	ALIC	3.21	− 22	22	8	28	7.14
	ACR	3.13	− 26	18	20	23	2.66
	Inferior frontal G_PT_	3.47	− 36	14	24	21	0.64
	PCR	3.55	− 20	− 22	22	20	4.48
	Putamen	3.39	− 28	− 14	10	19	0.58
	SFO	3.21	30	6	24	18	32.73
	Middle frontal G	3.47	− 32	12	38	18	0.55

Voxel-based lesion symptom mapping (VLSM) (lesion data of left hemisphere damaged patients flipped to the right to construct a joined dataset, n = 33).

Results for SLD—F and SLD—E (F, E = Flexion, Extension) are based on P_FDR_ < 0.05, corresponding to Z scores of 2.222 and 2.019 respectively. VLSM results for MT-F and NoP-E, as follows, did not survive FDR correction for multiple comparisons and are based on a more lenient criterion of p < 0.005 (corresponding to z ≥ 2.6): MT-F—middle occipital G (z value = 2.78, x–y–z =  − 28, − 74, − 2, 14 voxels, 0.43%area); NoP-E—insula (z-value = 2.93, x,y,z =  − 32,22,14, 16 voxels, 0.86%area). Other voxel-wise lesion effects were not significant.

*MT* movement time, *NoP* number of peaks, *SLD* straight line deviation, *F* flexion, *E* extension, *G* gyrus, *SCR/ACR/PCR* superior/anterior/posterior corona radiate, *PLIC/RLIC/ALIC* posterior/retro-lenticular/anterior limb of internal capsule, *PO/PT* pars opercularis/triangularis of inferior frontal gyrus, *SLF* superior longitudinal fasciculus, *EC* external capsule, *IFO/SFO* inferior/superior fronto-occipital fasciculus.

**Table 4:** Significant lesion effects on ‘stimulation gain’.

Condition	Structure	Z-value	x	y	z	Voxels	Area %
A-tDCS							
Movement time—E	Rolandic operculum	2.51	− 40	6	12	62	6.26
	SLF	3.01	− 36	− 20	32	44	5.4
	Insula	2.51	− 38	6	10	31	1.67
	Inferior frontal G_PO_	2.94	− 34	10	24	30	2.89
	Precentral G	2.51	− 44	6	18	27	0.77
	SLF	2.94	− 34	10	24	20	2.45
Number of peaks—E	SLF	2.41	− 32	− 10	36	14	1.72
Straight line deviation—E	PLIC	3.41	− 18	− 12	10	37	7.76
	Putamen	2.73	− 28	− 2	6	20	1.98
C-tDCS							
Number of peaks—E	Putamen	3.02	− 28	− 4	4	38	3.77
	Putamen	3.10	− 22	0	10	31	3.07
	EC	2.88	− 32	− 2	2	28	6.22
	IFO	2.51	− 34	4	− 4	16	6.61
Straight line deviation—E	ACR	3.53	− 24	20	20	91	10.52
	Insula	3.11	− 26	26	14	22	1.18
Straight line deviation—F	Insula	3.15	− 28	20	10	24	1.29
	ACR	3.17	− 24	20	10	22	2.54
	ALIC	3.17	− 22	18	10	15	3.83
	Putamen	3.17	− 22	16	10	14	1.39

Voxel-based lesion symptom mapping (VLSM) (lesion data of left hemisphere damaged patients flipped to the right to construct a joined dataset, n = 33). ‘Stimulation gain’ here means smaller NoP, shorter MT and smaller SLD in post- compared to pre-stimulation test, irrespective of whether this ‘gain’ reached significance in the ANOVA testing for differences between the 3 stimulation conditions (Table 2).

*F* flexion, *E* extension, *G* gyrus, *ACR* anterior corona radiate, *PLIC/ALIC* posterior/anterior limb of internal capsule, *PO* pars opercularis of inferior frontal gyrus, *SLF* superior longitudinal fasciculus, *EC* external capsule, *IFO* inferior fronto-occipital fasciculus.

The results presented here did not survive FDR correction for multiple comparisons, and based on a more lenient criterion of p < 0.005, reflecting z ≥ 2.6

## Results

### Differences between stroke patients and healthy subjects at baseline

The number of peaks (NoP) in flexion movements was significantly larger in stroke patients compared to healthy controls (2-tailed Mann–Whitney U test: U = 400.5, z = − 2.96, p = 0.003), with a median value of 3 and mean rank of 44.18 for stroke patients (n = 35) as compared to a median value of 2 and mean rank of 29.63 for healthy subjects (n = 35; the median value here is higher than expected for healthy subjects, and it stems from hesitant performance by a few subjects). In extension movements, the two-tailed Mann–Whitney U test also showed significantly larger NoP for the stroke group (U = 354, z =  − 3.35, p = 0.001) with a median value of 2.5 and mean rank of 44.43 in the stroke group as compared to a median value of 1.5 and mean rank of 28.11 in healthy controls.

Movement time (MT) in flexion was significantly longer in stroke patients compared to healthy controls (independent Student’s t-test: t_(70)_ = − 2.4, p = 0.016), with a mean MT of 1.6 ± 1.04 s in stroke patients, compared to 1.14 ± 0.46 s in healthy subjects. In extension, MT was also longer in the stroke group (t_(70)_ =  − 2.5, p = 0.019) with a mean MT of 1.63 ± 0.94 s in the stroke group compared to 1.2 ± 0.45 s in healthy controls.

Deviation from the straight line (SLD) in flexion movement was larger in the stroke group (independent Student’s t-test: t_(71)_ =  − 6.2, p < 0.001) with mean SLD of 0.55 ± 0.26 cm in the stroke group, as compared to 0.26 ± 0.08 cm in healthy controls. In extension movement the SLD was also larger in the stroke group (t_(71)_ =  −  3.4, p = 0.001), with a mean SLD of 0.65 ± 0.59 cm in the stroke group and 0.30 ± 0.09 cm in healthy subjects.

### Immediate effects of tDCS on end-point kinematics

The results of a mixed-design ANOVA with *Group* (Stroke, Control) as between-subjects factor and *Stimulation mode* (A-tDCS, C-tDCS, S-tDCS—all relate to the lesioned hemisphere in the patient group, or the left hemisphere in the healthy subjects) as within-subjects factor, are presented in Table 2.

As can be seen in Table 2, there was a significant main effect of *Stimulation mode* on gains revealed in NoP and MT measures. This was shown in arm movements employing elbow flexion but not elbow extension. The effect of *Stimulation mode*, both on the NoP gain and on the MT gain, emerged from a larger gain in A-tDCS compared to S-tDCS. The interaction of *Group* by *Stimulation Mode* reached significance in the NoP measure, as the above advantage of A-tDCS over S-tDCS was revealed in the stroke group but not in healthy subjects. As the origin of *Stimulation Mode* effects was in the stroke group, we performed a second analysis of the immediate effects of tDCS on arm kinematics, restricted to the stroke group. Friedman’s ANOVA of tDCS immediate effects on NoP, restricted to the stroke group, revealed no effect of stimulation mode in flexion movements (χ^2^ (0.95, 2) = 5.51, p = 0.07). Wilcoxon pairwise comparisons showed a significant difference between A-tDCS and S-tDCS: z = − 2.395, p = 0.01. The effect of stimulation mode on NoP in extension movements was not significant. One-way RM ANOVA of tDCS immediate effects on MT, restricted to the stroke group, also revealed an effect of stimulation mode in flexion movements: F(2,68) = 3.63, p = 0.039. Post-hoc analysis of MT results contrasting A-tDCS and S-tDCS conditions did not survive the Bonferoni correction for multiple comparisons (A-tDCS [M = 0.27 ± 0.48 s]; S-tDCS [M = − 0.02 ± 0.61 s], p = 0.07). The effect of stimulation mode on MT in extension movements was not significant. One-way RM ANOVA of stimulation mode effect on SLD, restricted to the stroke group and to flexion movements, did not reach the significance level (F(2,68) = 2.88, p = 0.06).

### Impact of baseline Fugl–Meyer scores on tDCS effects

**Figure 3:**
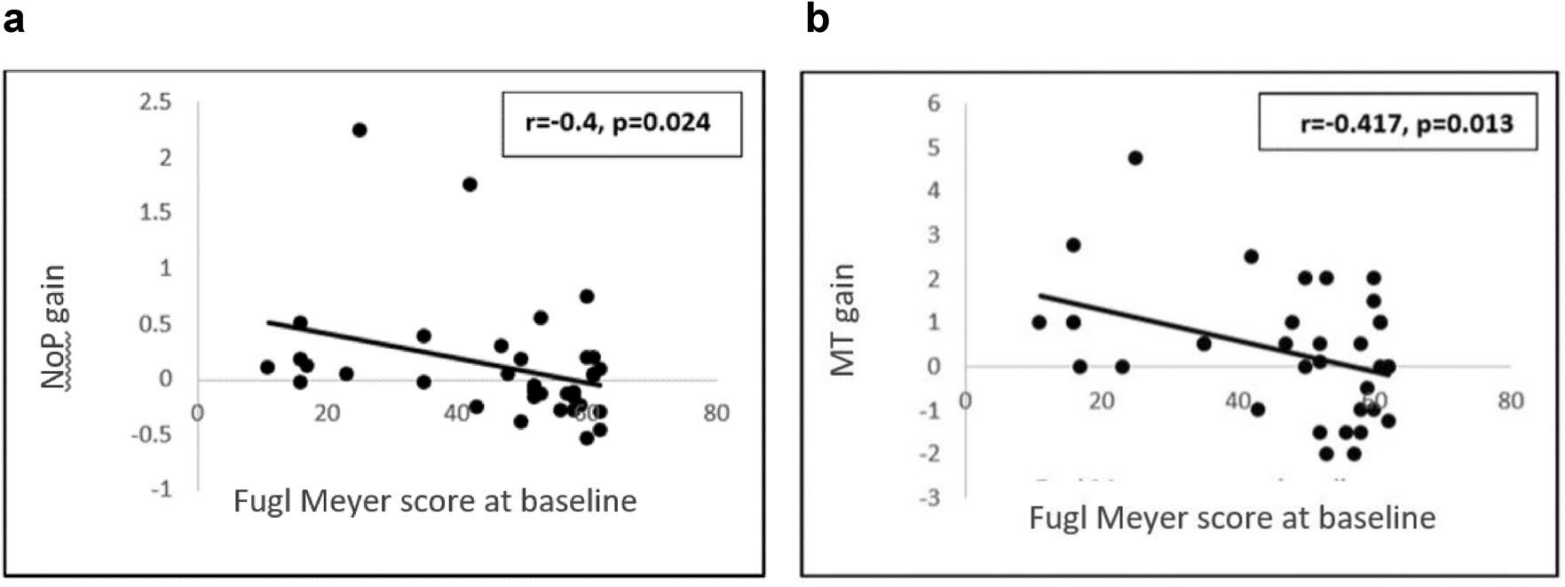
Correlation between stroke patients’ scores in the Fugl–Meyer assessment scale at baseline and the impact of a single C-tDCS session on reduction (improvement) in (**a**) number of peaks (NoP) and (**b**) movement time (MT). In the C-tDCS condition, the anode (excitatory stimulation) is placed over the primary motor cortex of the non-lesioned hemisphere. Lower baseline Fugl–Meyer scores, presumably indicating lower corticospinal reserve, were associated with higher gain in this condition, as reflected both in NoP and MT kinematic measures.

Spearman’s correlation analysis revealed a larger gain in stroke patients with lower baseline Fugl–Meyer scores (presumably, lower corticospinal reserve) compared to patients with higher baseline Fugl–Meyer scores (presumably, higher corticospinal reserve) in conditions of C-tDCS (i.e., with excitatory anodal stimulation applied on the non-affected hemisphere). This was shown in the form of significant negative correlation between baseline FMA and gain in this stimulation mode in NoP (r =  −  0.4, p = 0.024) and MT (r =  − 0.417, p = 0.013). (Fig. 3). The gain expressed in the SLD measure did not correlate with the baseline FMA in this stimulation mode. Correlation analyses between baseline FMA scores and gains in the two other stimulation conditions (A-tDCS, S-tDCS) were non-significant for all three kinematic measures.

### Impact of lesion characteristics on movement kinematics at baseline

Due to the relatively small cohorts of LHD and RHD patients, the left hemisphere was flipped to the right (as done in various earlier lesion studies, e.g.^44,45^) such that VLSM ignored lesion side. The analysis of lesion impact on the accuracy component of movement kinematics at baseline (SLD), exhibited a negative impact of damage to (a) white matter association and projection tracts (superior longitudinal fasciculus [SLF], inferior and superior fronto-occipital fasciculi, corona radiata, the external capsule and the different parts of the internal capsule); (b) cortical regions (insula, pre- and post-central gyri, rolandic operculum, inferior and middle frontal gyri, supramarginal and angular gyri of the inferior parietal cortex, superior and middle gyri of the temporal lobe, and to a lesser extent, more posterior parts of the occipital and occipito-temporal cortex; (c) putamen, caudate and thalamus. Damage to the above brain regions negatively affected SLD both in extension and flexion movement directions, with extension being affected by a larger extent of damage. The negative impact of brain damage on movement speed and smoothness (MT and NoP kinematic variables, respectively) did not survive the FDR correction for multiple comparisons. Using a more lenient criterion (p < 0.005, corresponding to z ≥ 2.6), MT was found to be affected by damage to the middle occipital gyrus, and NoP by damage to the Insula. The full description of VLSM results is shown in Table 3.

### Impact of lesion characteristics on immediate tDCS effects

Here also, due to the small size of the hemispheric cohorts we analyzed lesion effects after flipping the LHD data to the right, such that VLSM ignored lesion side. The analyses of lesion impact on the immediate effects of A-tDCS and C-tDCS (Table 4) did not survive the FDR correction for multiple comparisons. Using a more lenient criterion (p < 0.005, corresponding to z ≥ 2.6), the following lesion effects on immediate gain from A-tDCS reached significance: MT gain in extension was negatively affected by damage to (a) white matter association tract (superior longitudinal fasciculus); (b) cortical regions (rolandic operculum, insula, inferior frontal and pre-central gyri). NoP gain in extension was negatively affected by damage to the SLF, and SLD gain in extension was negatively affected by damage to the posterior limb of the internal capsule and the putamen.

Immediate gain from C-tDCS (excitatory stimulation of the contra-lesional hemisphere) was negatively affected by brain damage as follows: NoP in extension was affected by damage to the putamen, external capsule and the inferior fronto-occipital fasciculus. SLD in extension was affected by damage to the anterior corona radiata and insula, and SLD in flexion was affected by damage to the insula, anterior corona radiata, anterior limb of the internal capsule and the putamen.

In this section, the term ‘gain’ means smaller NoP, shorter MT and smaller SLD in the post- compared to the pre-stimulation test, irrespective of whether this ‘gain’ reached or did not reach significance in the ANOVA (Table 2) testing for differences between the three stimulation conditions.

**Figure 4:**
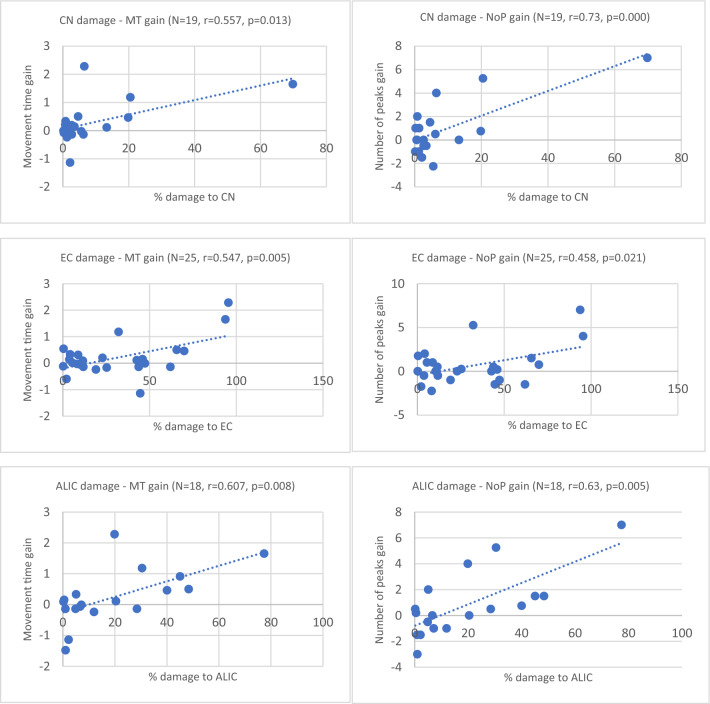
Brain structures in which the extent of damage correlated significantly with kinematic gain in the C-tDCS condition (excitatory anodal stimulation of the contralesional hemisphere. *CN* caudate nucleus, *EC* external capsule, *ALIC* anterior limb of internal capsule, *MT* movement time, *NoP* number of peaks. Despite measures undertaken to reduce the impact of outliers (see “Methods” section), given the limited number of data points in each analysis, it is impossible to eliminate decisively the possibility of a spurious correlation in some of these analyses^46,47^.

In a different approach, we analyzed the impact of damage within pre-defined regions of interest in the motor network on the immediate gain from C-tDCS (excitatory stimulation of the contra-lesional hemisphere), by correlating the extent of damage to these regions with the magnitude of negative effect on stimulation gain. The extent of damage within three brain structures—caudate nucleus, external capsule and anterior limb of the internal capsule—correlated negatively in a significant manner with C-tDCS gain, as reflected in MT (speed) and NoP (smoothness) of reaching movements in the paretic upper limb (Fig. 4). Other correlations did not reach significance. (Supplementary Fig. 1 shows normalized lesion data of individual patients and Supplementary Table 1 shows pre- and post-stimulation average group performance).

## Discussion

When considering the use of tDCS, or other modalities of NIBS, in the rehabilitation plan of an individual stroke patient, it is crucial to know whether the patient is likely to benefit more from up-regulation of the motor system in the lesioned or the non-lesioned hemisphere, as wrong target selection may lead to null or even detrimental effects^17–20^.

In this study, we asked whether variables derived from the kinematic analysis of reaching movement by the hemiparetic upper limb, are sensitive enough to capture differential neuro-modulatory effects of tDCS applied over the motor cortex in the lesioned versus the non-lesioned hemisphere. Specifically, whether such differential effects can be shown immediately following a single session of reaching training combined with tDCS. Suppose such differential effects can be shown. In that case, they are expected to help expand the effective use of treatments employing tDCS, by informing on the brain side where stimulation is more likely to facilitate recovery in individual patients. In the future, with the accumulation of information from larger groups of patients, such immediate effects on motor performance, combined with corticospinal lesion-load data, and corticospinal transmission data (from MEP studies), may help structuring comprehensive algorithms aimed to support clinical decision making in this respect^48^.

First, we verified that the chosen kinematic descriptors capture the disadvantage of stroke patients compared to healthy controls. Indeed, members of the stroke group performed the reaching movements significantly more slowly, less accurately, and with diminished smoothness (evidenced by MT, SLD and NoP results, respectively), compared to members of the control group, both in flexion and extension arm movements. This finding is in accord with a previous stroke study that showed movement time to be longer in the paretic arm compared to the other arm and compared to healthy control subjects^49^. In another study, several peaks were present in the trajectory of the paretic upper limb, as opposed to the smooth bell-shaped velocity profiles of the non-paretic limb, and the path length of the reaching trajectory was increased for stroke patients compared to healthy control subjects^50^. Thus, the results of the first part of our study attest to the sensitivity of the selected kinematic descriptors (MT, SLD and NoP) to demonstrate different aspects of motor impairment of the hemiparetic upper limb, as reflected in point-to-point reaching movements. Shortening of MT, reduction of SLD, and reduction of NoP can thus be taken as indicators of treatment-induced recovery.

In the second part of the study, we examined how these three kinematic variables are affected by a single session of reaching training conducted in conjunction with tDCS (120 repetitions of the reaching task undertaken during the electrical stimulation). As in each treatment session, a different tDCS montage was used, the design enabled comparative assessment of the immediate impact of A-tDCS versus C-tDCS versus S-tDCS on performance gain from a single training session.

In the healthy control group, there was naturally less room for improvement, and task performance disclosed quite similar movement kinematics before and after the single treatment session, with insignificant stimulation-mode effects. Different tDCS past studies also failed to demonstrate, in healthy subjects, clear neuro-modulatory effects, or effects of electrode montage, on performance level in different motor tasks^51–53^, though in a study with greater accuracy requirements, which examined effects of high-definition tDCS on movement quality in healthy subjects, minor improvement in the MT variable, but not in other kinematic variables, has been observed^54^. Our stroke patients had more room for improvement, and two of the three kinematic variables—NoP (movement smoothness as expressed by the number of peaks in the endpoint velocity profile) and MT (speed)—showed significant stimulation-mode effects. Analysis of variance and post-hoc analyses revealed differential effects restricted to the stroke group, where excitatory stimulation of the ipsilesional hemisphere (A-tDCS condition) had a greater positive impact on movement kinematics compared to sham stimulation (S-tDCS condition). Based on the bimodal balance recovery model^18^ we may assume that in this specific cohort of stroke patients (average FMA 46.6 ± 16.3) there were more patients likely to benefit from up-regulation of the lesioned hemisphere and enhancement of transmission through the crossing CST (A-tDCS condition), than patients likely to benefit from up-regulation of the non-lesioned hemisphere and enhancement of transmission through the contralesional non-crossing segments of the CST and RST (C-tDCS condition). This can explain the manifestation of A-tDCS (but not C-tDCS) advantage over the sham condition. Also, down regulation of the lesioned hemisphere in the C-tDCS condition might have reduced the training effect, making it closer to the sham condition.

In the stroke group, the effects of stimulation mode, both on NoP and on MT, reached significance in movements involving elbow flexion but not extension. This finding probably reflects greater residual range of active movement and room for improved kinematics in movements undertaken as part of the flexor synergy, which is frequently encountered in the hemiparetic upper limb of stroke survivors. In these patients, reaching movements involving elbow extension require more effort to overcome the spasticity of flexor muscles and are slower and less accurate compared to reaching movements involving elbow flexion. The latter utilize components of the flexor synergy, whereas elbow extension involves breaking out of the flexor synergy^55,56^.

While stroke patients revealed an effect of stimulation-mode on NoP (greater reduction of NoP under A-tDCS compared to S-tDCS), such an effect was not obtained among the healthy subjects. Looking at the velocity profile of healthy subjects, we noted that 37% of them exhibited, already at the pre-stimulation phase, the optimal pattern for planar point-to-point movement, of a single velocity peak (just one acceleration-deceleration transition within the movement trajectory^27^) compared to only 8% of the stroke patients who exhibited this pattern. Thus, already at the pre-stimulation stage, over one third of the healthy subjects had no room for further improvement in the smoothness variable. Note that stimulation mode had an effect also on MT in the stroke group. Reduction of movement time usually yields, in itself, a smoother movement with less fragmentation, i.e. fewer NoP^57^. Thus, NoP reduction here may relate to MT shortening and only indirectly to the effect of stimulation-mode (A-tDCS advantage).

The third kinematic variable—extent of deviation from the straight line (SLD)—representing movement accuracy, showed no effect of stimulation mode, neither in the control nor in the stroke group. The lack of stimulation-mode effect on this kinematic descriptor can be interpreted as a sign that a single session of reaching training combined with tDCS does not modulate the brain mechanisms ensuring movement accuracy, or that the measure we used (SLD) is not sensitive enough to capture such modulation. It may also be related to the fact that MT and NoP (the kinematic descriptors that exhibited stimulation-mode effects) are temporal measures, whereas SLD (which did not exhibit such effects) is a spatial measure. Theoretical modeling and empirical evidence point to distinct neural organization of spatial and temporal aspects of motor planning, with spatiotemporal integration occurring towards the execution of the motor act^58–60^. The manifestation of stimulus-mode effects on measures of speed and smoothness (MT, NoP) but not on the measure of accuracy (SLD) may also relate to the instruction given to ‘move as fast and as accurate as possible’. Although no feedback was given for any of the instruction components, it may be easier to adhere to ‘move fast’ than to ‘move accurately’ and often moving faster results in lower accuracy (speed-accuracy tradeoff).

FMA score at baseline (taken as an indicator of impairment level) showed a negative correlation with the gain reveled in the NoP and MT measures (of movement smoothness and speed, respectively) under C-tDCS conditions (cathode over ipsilesional M1, anode over contralesional M1). This may indicate that for patients exhibiting severe impairment level post stroke (presumably pointing to greater damage to corticospinal transmission from the lesioned hemisphere^48^), excitatory stimulation of the non-affected hemisphere is likely to be more beneficial than to patients with milder impairments (yet, this inference should be taken with caution, as the correlation between the FMA score at baseline and the gain revealed in the accuracy measure (SLD) was not significant). How does the finding of a significant negative correlation between impairment level and performance gain, shown selectively under C-tDCS conditions, relate to the theoretical assumptions that motivate and guide tDCS treatments? The inter-hemispheric competition model posits the existence of reciprocal inhibitory influences between the two hemispheres. According to this model, impairment caused by structural damage in one hemisphere is aggravated by an inhibitory influence coming from the non-involved hemisphere. The negative impact of this inhibitory effect is accentuated because the hemisphere affected by the stroke fails to deliver a normal counterbalancing reciprocal inhibition. Based on this theorizing, and on physiological evidence thought to support it^14^, a large number of clinical trials that used tDCS to enhance recovery post stroke, aimed to up-regulate the lesioned hemisphere by excitatory anodal stimulation, and/or to down-regulate the non-lesioned hemisphere by inhibitory cathodal stimulation. However, an extensive literature review^61^ found no satisfactory evidence that use of tDCS in this form benefits upper-limb function post stroke. The large variance shown in the results of reviewed studies (which led to this disappointing conclusion) is thought to reflect uncontrolled variance in the extent to which re-mapping in the lesioned hemisphere can support functional recovery. Another theoretical proposition—the bimodal balance-recovery model^18^—posits that NIBS should aim to up-regulate the hemisphere which is more likely to support functional recovery. In cases of extensive unilateral destruction of the motor system, re-instatement of cerebral control over spinal motor activity—a prerequisite for restitution of voluntary movement in the paretic upper limb—may depend on the contra-lesional rather than on the lesioned hemisphere. In such cases, NIBS should aim to up-regulate non-crossing corticospinal and reticulospinal tracts descending from the contra-lesional hemisphere^33^. Our demonstration here that the C-tDCS montage (up-regulation of the non-involved hemisphere) exerts an immediate positive effect on movement quality, as expressed in the NoP and MT kinematic descriptors, is in accord with the notion of a positive role for the non-damaged hemisphere in motor recovery post stroke, at least in part of the patients. Moreover, the fact that NoP gains and MT gains in the C-tDCS condition maintained a significant negative correlation with patients’ baseline FMA scores, is in accord with the bimodal balance-recovery model^18^, as it shows that a positive outcome from up-regulation of the non-damaged hemisphere is more likely to occur in the more severely impaired stroke patients.

What is the impact of lesion topography on the likelihood of a patient to benefit from tDCS neuro-modulation? We used voxel-based lesion-symptom mapping (VLSM) to explore first the effect of lesion topography on movement kinematics at baseline, and then its effect on treatment gains, as reflected in the three descriptors of end-point kinematics. Movement accuracy (SLD) at baseline was affected by damage to white-matter association and projection tracts, different regions in the lateral aspect of the cortical mantle, the basal ganglia and the thalamus. The impact of brain damage on movement speed and smoothness (MT and NoP variables, respectively) did not survive the FDR correction for multiple comparisons. VLSM analyses of lesion impact on treatment gains yielded effects only when a lenient criterion for significance (p < 0.005, un-corrected) was used. The immediate gain in movement speed (MT), from a single session of reaching training in conjunction with A-tDCS, was affected by damage to the superior longitudinal fasciculus (SLF) and to a few regions in the lateral aspect of the cortical mantle. In this condition (A-tDCS), the gain revealed in movement smoothness (NoP) was affected by damage to the SLF, and the gain revealed in movement accuracy (SLD) was affected by damage to the posterior limb of the internal capsule and the putamen (all these effects appeared in extension movements). Gain from C-tDCS, as reflected in movement smoothness (NoP), was affected by damage to the putamen, external capsule and the inferior fronto-occipital fasciculus. SLD gain in extension was affected by damage to the anterior corona radiata and insula, and SLD gain in flexion was affected by damage to the insula, anterior corona radiata, anterior limb of the internal capsule and the putamen. The extent of tissue damage within three brain structures—caudate nucleus, external capsule and anterior limb of internal capsule—correlated negatively with C-tDCS gain, as reflected in MT (speed) and NoP (smoothness). Other correlations did not reach significance. Note that each descriptor of movement kinematics was affected by damage to a different set of brain structures under A-tDCS versus C-tDCS (e.g., NoP gain in extension was affected by damage to the SLF under A-tDCS and by damage to the putamen, external capsule and inferior fronto-occipital fasciculus under C-tDCS). The lesion effects reported here refer to ‘gains’ revealed in the form of smaller NoP, shorter MT or smaller SLD in the post- compared to the pre-stimulation test, irrespective of whether these ‘gains’ reached or did not reach significance in the ANOVA testing for differences between the three stimulation conditions (Table 2). Thus, the VLSM results presented here reflect the impact of variation in stroke lesion topography on response variability under the different stimulation conditions.

Un-solved theoretical issues concerning tDCS effects preclude, at the current state of knowledge, a conclusive interpretation of some of the current findings. Although the anode and cathode poles in tDCS are known to exert excitation and suppression of corticospinal transmission, respectively^62,7^, the physiological reality of tDCS neuro-modulation seems to be more complicated, especially in the case of a bi-hemispheric montage, as applied in the current study. Both, the exact mechanism and the relative efficacy of tDCS effects in uni-hemispheric and bi-hemispheric montages awaits clarification^8,63–67^. Polarity-specific excitability changes are reduced in the bi-hemispheric montage^68^ and some studies have shown that regardless of whether the anode is placed over the contralateral or ipsilateral motor cortex, bi-hemispheric stimulation yields substantial performance gains relative to uni-hemispheric or sham stimulation, with no significant difference between the two bi-hemisphere montages^51,69^. These findings cast doubt on whether our interpretation of gains shown in the C-tDCS condition, based on the bimodal balance-recovery model, is the sole or even the more likely interpretation.

### Limitations of the study

1. Given the moderate cohort size, we had to conduct lesion-symptom analyses after flipping left-sided lesion data to the right, such that our VLSM results ignore lesion side. Although this procedure was employed in various past studies in order to increase statistical power (e.g.^44,45^), it undeniably masks important effects, as shown in our own earlier stroke studies^46,70^.
2. The combination of large inter-personal variance and moderate cohort size resulted in attenuated statistical power. This left a measure of uncertainty with respect to the biological reality underlying some of the effects shown in this study. This applies (a) to the finding that advantage of anodal over sham stimulation of ipsilesional M1 was shown only for the temporal kinematic variables (MT, NoP) but not for the spatial variable (SLD); (b) to the finding that gain from A-tDCS applied on the non-lesioned hemisphere correlated with pre-stimulation impairment level, whereas the expected corollary negative correlation between the gain from A-tDCS applied on the lesioned hemisphere and pre-stimulation impairment level was not shown; (c) to the finding that SLD was the only kinematic variable to exhibit significant (P_FDR_ < 0.05) effects of brain damage at the voxel level. Further research will be needed to provide more conclusive assertions on these findings.
3. Studies based on moderate-size cohorts of un-stratified stroke patients (as in the current research and in the large majority of past tDCS studies) are probably incapable of clarifying conclusively the mechanisms underlying the large response variability in treatments employing tDCS, and NIBS in general. The remaining uncertainty is responsible for the fact that interventions employing NIBS did not become part of the standard clinical practice in stroke rehabilitation. The results of the current study point to characteristics of lesion topography, and to baseline impairment level, as factors contributing in a specific direction to response variability. The results also point to the potential of kinematic variables derived from a single treatment session to inform on relative advantage of different tDCS montages. However, the clinician’s question—how to decide on the optimal target location for tDCS, for the individual stroke patient—awaits further research.
4. The current study contrasted two versions of a bilateral montage condition: [il-M1 A-tDCS, cl-M1 C-DCS] vs. [il-M1 C-tDCS, cl-M1 A-tDCS], plus a third sham condition. However, as the physiological effect of the cathode pole in neuromodulation is currently far from clear ^8^, and the key issue relates to placement of the anode pole (to verify which hemisphere should be up-regulated by excitatory stimulation to optimally support the recovery process in a stroke patient with a given measure of baseline motor impairment), future research aimed to clarify this question may better use unilateral anodal stimulation of il-M1 vs. cl-M1, with the cathode pole placed over the contralateral supraorbital region, rather than the bilateral montage.

## Conclusions

The results of this study show that simple descriptors of end-point kinematics (MT, NoP and SLD) derived from planar point-to-point reaching movements of the hemiparetic upper limb, are sensitive enough to demonstrate gain from just a single session of reaching training conducted with concomitant tDCS. Moreover, the pattern of gain shown by these kinematic descriptors is sensitive (a) to the montage of tDCS electrodes, i.e., to the added effect of brain stimulation, beyond the effect of the reaching training itself, (b) to the severity of baseline motor impairment, and (c) to the pattern of brain damage.

### Supplementary Information

Supplementary Information.

## Data Availability

Datasets generated during and/or analyzed during the current study are available from the corresponding author (NS) on reasonable request.
