# Supplementary material for: Kinematic descriptors of arm reaching movement are sensitive to hemisphere-specific immediate neuromodulatory effects of transcranial direct current stimulation post stroke
Source: Sci Rep. 2024 May 25;14:11971. doi: 10.1038/s41598-024-62889-0 (PMC11127956; doi:10.1038/s41598-024-62889-0)
Supplement: Supplementary file 1 — Supplementary Information. [file 41598_2024_62889_MOESM1_ESM.docx]

Supplementary information to:

Justine Lowenthal-Raz, Dario G. Liebermann, Jason Friedman and Nachum Soroker. *Kinematic Descriptors of Arm Reaching Movement are Sensitive to Hemisphere-Specific Immediate Neuromodulatory Effects of Transcranial Direct Current Stimulation Post Stroke*.

**Supplementary Table 1: Pre- and post-stimulation average group performance**

| variable | Movement  direction | Stimulation  mode | Controls*  Pre-stimulation  Mean (SD) | Controls*  Post-stimulation  Mean (SD) | Stroke  Pre-stimulation  Mean (SD) | Stroke  Post-stimulation  Mean (SD) |
| --- | --- | --- | --- | --- | --- | --- |
| NoP | Flexion | A-tDCS | 2.22 (1.60) | 1.92 (1.16) | 3.10 (1.89) | 2.27 (1.27) |
|  |  | C-tDCS | 2.11 (1.41) | 1.82 (0.98) | 3.02 (1.42) | 2.66 (1.63) |
|  |  | S-tDCS | 2.13 (1.25) | 1.95 (1.18) | 2.78 (1.56) | 3.02 (2.11) |
|  | Extension | A-tDCS | 1.19 (1.23) | 1.75 (1.31) | 3.28 (3.59) | 2.41 (1.60) |
|  |  | C-tDCS | 1.74 (0.88) | 1.73 (0.90) | 3.07 (2.11) | 2.67 (1.56) |
|  |  | S-tDCS | 1.70 (0.84) | 1.72 (0.80) | 2.88 (1.92) | 3.02 (1.88) |
| MT  (sec) | Flexion | A-tDCS | 1.16 (0.45) | 1.10 (0.47) | 1.55 (0.65) | 1.27 (0.38) |
|  |  | C-tDCS | 1.08 (0.43) | 1.00 (0.38) | 1.53 (0.69) | 1.41 (0.49) |
|  |  | S-tDCS | 1.15 (0.44) | 1.07 (0.40) | 1.49 (0.52) | 1.52 (0.62) |
|  | Extension | A-tDCS | 1.23 (0.47) | 1.15 (0.51) | 1.62 (0.93) | 1.35 (0.51) |
|  |  | C-tDCS | 1.17 (0.43) | 1.10 (0.46) | 1.62 (0.65) | 1.46 (0.59) |
|  |  | S-tDCS | 1.17 (0.47) | 1.18 (0.45) | 1.56 (0.82) | 1.56 (0.71) |
| SLD  (cm) | Flexion | A-tDCS | 0.26 (0.06) | 0.28 (0.09) | 0.57 (0.27) | 0.50 (0.26) |
|  |  | C-tDCS | 0.27 (0.10) | 0.28 (0.08) | 0.53 (0.35) | 0.57 (0.46) |
|  |  | S-tDCS | 0.27 (0.07) | 0.28 (0.10) | 0.51 (0.43) | 0.54 (0.32) |
|  | Extension | A-tDCS | 0.30 (0.12) | 0.30 (0.13) | 0.58 (0.28) | 0.54 (0.23) |
|  |  | C-tDCS | 0.28 (0.09) | 0.28 (0.10) | 0.60 (0.39) | 0.58 (0.30) |
|  |  | S-tDCS | 0.31 (0.10) | 0.28 (0.08) | 1.03 (2.51) | 0.58 (0.31) |

* Number of controls in flexion movement: n=32, in extension movement: n=34 (the different n results from exclusion of outliers, i.e., subjects with very unusual performance, deviating by more than 2 SD from the group average); NoP = number of peaks; MT = movement time; SLD = straight line deviation; A-tDCS = Anode placed over the hand area of the primary motor cortex in the lesioned hemisphere; C-tDCS = Cathode placed over the hand area of the primary motor cortex in the lesioned hemisphere (homologous region in the non-lesioned hemisphere is under the anode); S-tDCS = Sham stimulation. For healthy controls, the three stimulation modes refer to the left hemisphere.

**Supplementary Figure 1:** Normalized lesion data. Each patient's lesion marked on arrays of 11 standard templates (Damasio and Damasio, 1989). Displays follow neurological convention, i.e., right sided damage displayed on the left and left sided damage displayed on the right side. Only slices that present brain damage are shown. One patient from the RHD group does not appear due to small lesion size of 0.32cc.

**
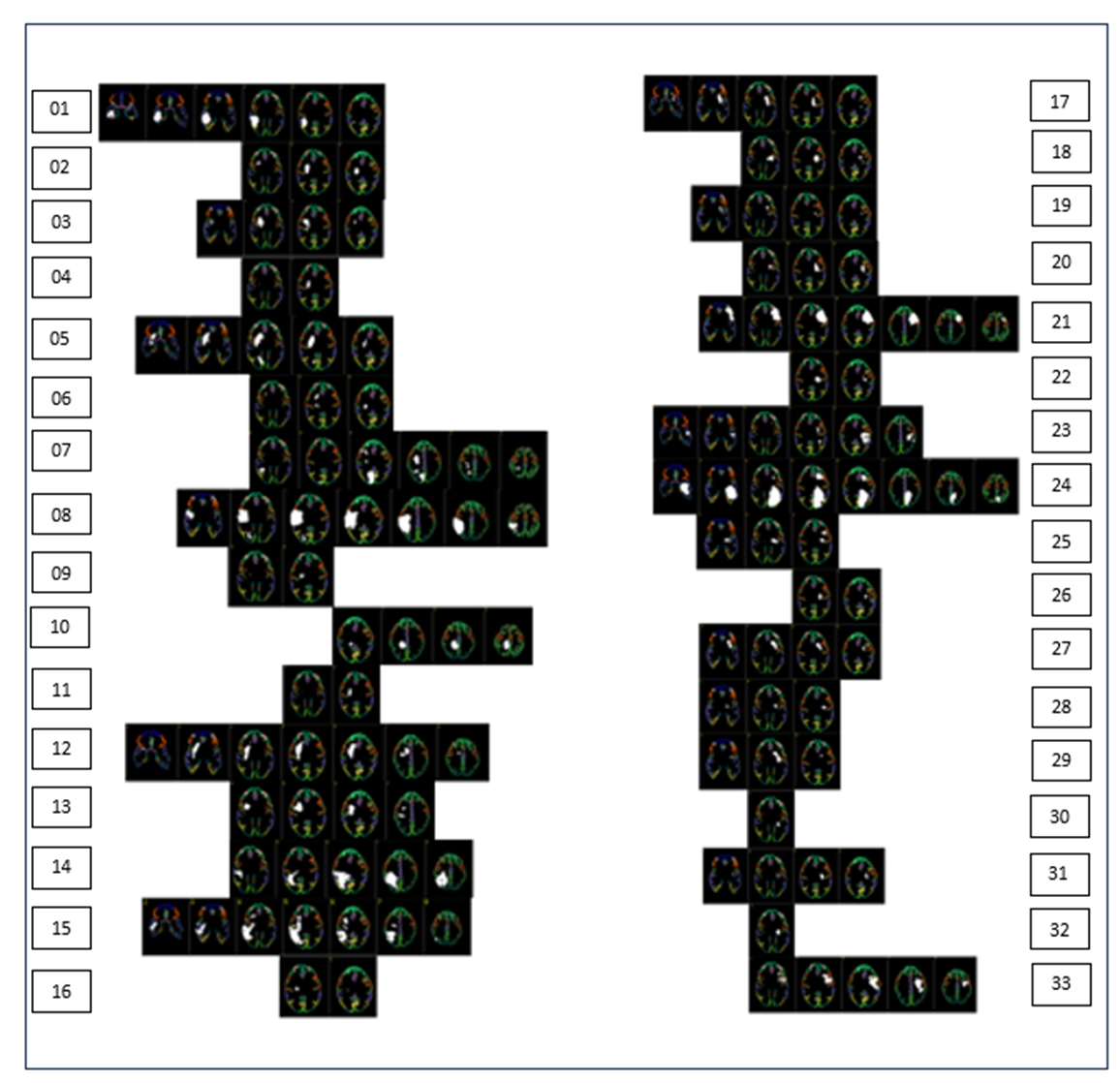
**
